# 100 years of Haldane's rule

**DOI:** 10.1111/jeb.14112

**Published:** 2022-11-10

**Authors:** Finn Cowell

**Affiliations:** ^1^ School of Biological Sciences, University of St Andrews St Andrews UK

**Keywords:** dominance theory, dosage compensation, faster‐male theory, faster‐X theory, Haldane's rule, hybrid sterility, meiotic drive, postzygotic isolation, sex chromosomes, speciation

## Abstract

Haldane's rule is one of the ‘two rules of speciation’. It states that if one sex is ‘absent, rare or sterile’ in a hybrid population, then that sex will be heterogametic. Since Haldane first made this observation, 100 years have passed and still questions arise over how many independent examples exist and what the underlying causes of Haldane's rule are. This review aims to examine research that has occurred over the last century. It seeks to do so by discussing possible causes of Haldane's rule, as well as gaps in the research of these causes that could be readily addressed today. After 100 years of research, it can be concluded that Haldane's rule is a complicated one, and much current knowledge has been accrued by studying the model organisms of speciation. This has led to the primacy of dominance theory and faster‐male theory as explanations for Haldane's rule. However, some of the most interesting findings of the 21st century with regard to Haldane's rule have involved investigating a wider range of taxa emphasizing the need to continue using comparative methods, including ever more taxa as new cases are discovered.

## Graphical abstract

Haldane's rule: when hybrids from one sex are absent, rare or sterile, that sex is always the heterogametic sex.

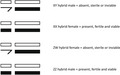

## 1 INTRODUCTION

The polymath J.B.S Haldane was a major populariser of science and contributed to a wide variety of discoveries relating to enzyme kinetics, biochemical genetics, physiology, biostatics and theories on the origin of life, among other topics (Dronamraju, 2010). Furthermore, Haldane's contributions to the field of population genetics, along with Sewall Wright and R.A. Fisher, were pivotal in the creation of the modern synthesis (Pirie, 1966).

An early contribution of Haldane's was a 1922 paper that noted the following: ‘When in the F1 offspring of two different animal races one sex is absent, rare or sterile, that sex is the heterozygous sex’. Haldane (1922) uses the term ‘heterozygous’ to refer to an individual's sex chromosomes differing. In modern parlance, the term ‘heterogametic’ is used, where X‐Y chromosomes are present in males of taxa such as Mammalia and Diptera and Z‐W chromosomes are present in females of taxa such as Aves and Lepidoptera. Haldane initially showed this rule to hold true in several animal taxa with sex chromosomes, and the expected pattern has since been shown to be almost ubiquitous throughout the animal kingdom, as well as having now been observed in plant species (Brothers & Delph, 2010; Delph & Demuth, 2016; Kasjanuk et al., 2019).

The year 2022 marks the hundredth anniversary of Haldane's observation making this an apt time to take stock of the history of research into what is one of the most widely conserved rules in evolutionary biology and still very much an active topic of research. This review will discuss the wide‐ranging evidence that has led most to consider Haldane's rule as an important principle in the study of speciation. The hypotheses that seek to explain the causes of the rule will then be explained and evaluated using examples from selected key studies.

## 2 IS HALDANE'S RULE REAL?

When Haldane first formulated the eponymous rule, it was done by looking at species crosses from four taxa: Mammalia, Aves, Lepidoptera and a single species pair of Diptera (Haldane, 1922). The number of Mammalian species was expanded by Craft (1938) and Coyne and Orr (1989a) compiled a list of *Drosophila* species that obey Haldane's rule. Expansions into other taxa followed and among taxa of interest, the evidence that Haldane's rule is statistically significant seems overwhelming. It holds true in 95% (*n* = 131) of *Drosophila* species, 100% (*n* = 26) of mammals, 97% (*n* = 87) of birds and 96% (*n* = 114) of Lepidopterans (Presgraves, 2008). Within the study of speciation, generalities such as this are rare owing to the local aspects of the environments inhabited by species and their history. This has led to Haldane's rule becoming known as one of the ‘two rules of speciation’, the other being the large‐X effect (Coyne, 2018; Coyne & Orr, 2004).

It may then come as a surprise that the statistical significance of Haldane's rule has in the past been questioned. Read and Nee (1991) made the case that sample sizes have been spuriously inflated by using the number of hybridisations between the different species within which Haldane's rule has been observed, rather than the number of times heterogamety has evolved in a taxon. For example, they looked at the number of insect species that had given rise to hybrids that follow Haldane's rule (which numbered 191 at the time) and argued that, rather than there being 191 relevant pieces of information, there were only two: one from the female heterogamety in the Z‐W systems and one from male heterogamety in the X‐Y systems. Read and Nee consider noting the instances of these independent evolutions to be important as, when the taxa differ, heterogamety is usually one of many differences. Birds and *Drosophila* for instance differ in their gametogenesis, embryogenesis and sex‐specific traits. It is therefore concluded that given the small number of independent origins of heterogamety (just four according to their phylogenetic analysis), then Haldane's rule does not have the statistical power to be called significant.

Building up the necessary statistical power by considering only truly independent data points is key to the comparative methods used in evolutionary biology, but it was also pointed out in a companion paper to Read and Nee's by Coyne et al. (1991) that genetic studies do show that sex chromosomes play an important role in the loss of fitness in hybrids. Indeed, this was the case for all genetic studies of Haldane's rule at the time (Coyne & Orr, 1989b). This important piece of evidence should be considered in any assessment of the biological significance of Haldane's rule.

Furthermore, Read and Nee omitted salamanders of the genus *Triturus*, which independently evolved male heterogamety, and additional independent origins are being documented (Orr, 1997). Tabulations of the independent origins of heterogametic sex now equal nine in the animal kingdom and the observation of the rule in the *Silene* genus of plants now brings the total to 10 independent origins (Delph & Demuth, 2016). Read and Nee therefore asked the important question of how independent the contrasts between species are and part of the response to this continues to be a growing list of independent origins of heterogamety in which Haldane's rule holds true.

It is also of course important to consider whether all members of a taxon really do constitute only one independent origin. For example, the differing composition of sex chromosomes might be a long‐lived sign of the evolution of heterogamety. Vicoso and Bachtrog (2015) showed that Dipterans have sex chromosomes that segregate differently, despite being an XY system. This, they show, is due to what are known as Muller elements: the common units that make up chromosomes, of which there are six (A‐F), five large rods and one small dot. They showed that, in Tipulidae, element F (the small dot) segregates as the X chromosome, while other elements are used in a variety of different ways for the X chromosome in other taxa within the Diptera order. What Read and Nee therefore described as a single case of heterogamety evolving independently may in fact represent multiple independent origins, depending on the mechanistic origins of Haldane's rule.

This does not mean that taxa with the same Muller elements have not evolved their chromosomes independently. Indeed, *Anopheles gambiae* mosquitos have an X chromosome that is independently derived from that of *Drosophila melanogaster*, and yet, they have the same Muller element comprising their X chromosome (Vicoso & Bachtrog, 2015). Instead, when the muller elements do differ this taxon can be confirmed as an independent case of heterogamety evolving, but it must be acknowledged that this will produce a conservative estimate and, inevitably, some more distantly related species with homologous chromosomes will remain unknown. These cases can then be used to produce a minimum estimate of the number of cases of independent origins of heterogamety. In their review on Haldane's rule, Delph and Demuth (2016) note that if one is to take this definition of independent origin, the total number can be increased from 10 to 15, so as to include the four independent origins in Diptera and two genera of stickleback.

## 3 POSTZYGOTIC ISOLATION

Some would consider the generality of Haldane's rule interesting in its own right. However, the driver of most of the research into this topic is the implications that it has for the process of speciation through postzygotic isolation. The importance of this form of isolation has been appreciated for some time with Dobzhansky (1957), in a book first published in 1937, noting several forms of isolating mechanisms that fit this description, including hybrid inviability, hybrid breakdown (both forms of inviability) and hybrid sterility. Hybrid dysfunction is therefore a long‐studied form of postzygotic isolation known as intrinsic postzygotic isolation, where developmental issues in hybrids lead to reproductive isolation in a way that is largely independent of the environment (Coyne & Orr, 2004). Coyne and Orr (1989a) demonstrate that there are two main ways a pair of species can evolve postzygotic isolation. One of these would involve the heterogametic sex being sterile or inviable, while the other would involve both sexes becoming sterile or inviable simultaneously. While Haldane's rule holds true under both scenarios, they found that the former is the more common in *Drosophila* species.

The importance of Haldane's rule to postzygotic isolation naturally leads to further questions about the underlying genetics. In a landmark paper, Dobzhansky (1936) looked at sterility in hybrids and attributed it to the interactions between genetic factors of both parents. Dobzhansky imagined one parent with an SStt genotype and another with ssTT. The resulting hybrid would be SsTt. S and T by themselves may be of no detriment to an individual, but if S and T are together in a hybrid, then this may cause sterility. Dobzhansky demonstrated experimentally that such interactions do occur, falsifying some of the previous hypotheses that claimed hybrid sterility was due to either cytoplasmic differences or chromosomal rearrangements that disrupted chromosome pairing in the hybrid (Orr, 1996).

Dobzhansky's discoveries also supplied some of the best evidence for what is today known as the Dobzhansky‐Muller model, owing to the independent work of these two scientists (Orr, 1997). It should be noted that this all‐important model should, in fairness, be called the Bateson‐Dobzhansky‐Muller model (BDM), because, in 1909, William Bateson presented an almost identical explanation of hybrid sterility in a long‐forgotten essay, rediscovered by Orr (1996). Muller (1942) meanwhile contributed to this model by showing that, in *Drosophila*, incompatibilities can often involve multiple interactions between genes, not just interactions between pairs of genes. Muller also theorized that these BDM incompatibilities form the basis for most explanations of Haldane's rule. Many review papers seek to only explain these more recent and plausible theories; however, the present review seeks to explore the history of research into Haldane's rule, and so, every important hypothesis will be explored, even if it is not currently dominating the literature (see Table 1 for a summary). It is also worth pointing out that it is unlikely that a single hypothesis can explain Haldane's rule; the causal mechanisms are likely different in different taxa.

**TABLE 1:** The different hypotheses that have been offered as explanations for Haldane's rule

Theory	Applicable to all sex determination systems?	Genetic explanation	Source
Sexual Transformation	Yes	Hybrids are transformed into the homogametic sex but remain chromosomally heterogametic.	Haldane (1922); Goldschmidt (1934)
Chromosomal Rearrangements	Yes	A piece of chromosome breaks off the Y or W chromosome and is relocated on another, causing an incompatibility.	Haldane (1932)
Y Incompatibilities	Yes	Y is incompatible with X chromosome or autosomes.	Muller (1942)
Dosage Compensation	Yes	Hybridisation causes problems with regulation and/or activation of the dosage compensation process needed in heterogametes.	Coyne and Orr (1989b)
Dominance	No	Recessive X‐ or Z‐linked genes cause sterility.	Muller (1942)
Faster‐Male	No	Faster divergence of male reproductive genes than female ones.	Wu and Davis (1993)
Faster‐X	Yes (but not a direct cause)	Faster divergence of X‐ or Z‐linked loci relative to autosomal ones.	Charlesworth et al. (1987)
Meiotic Drive	Yes	Discrepancy between drivers and suppressors on sex chromosomes leads to sterility.	Frank (1991); Hurst and Pomiankowski (1991)

## 4 THE CAUSES OF HALDANE'S RULE

### 4.1 Sexual transformation

Haldane (1922) considered that the lack of heterogametic individuals could be due to either their being killed off or due to their sexual transformation (where the heterogametic sex appears as the normally homogametic sex). Evidence of the latter came from two Lepidopteran genera: *Fumea* and *Lymantria*. Subsequent work from Goldschmidt (1934), claimed that half of all males from *Lymantria dispar* crosses were in fact fully transformed females, which are heterozygous. However, Clarke and Ford (1982), who performed the same crosses and then used sex chromatin techniques to identify the chromosome compliments, found that, although there was a large excess of males (as Haldane's rule would predict), these were also chromosomally male.

As pointed out by Laurie (1997), even if this explanation is correct, it cannot completely account for Haldane's rule as heterogametic hybrid inviability has been shown to occur in many cases. Nonetheless, Laurie called for more investigation into this potential explanation of Haldane's rule. Disappointingly, it would appear that very little research has occurred since 1997, although, in a cross between *Caenorhabditis briggsae* males and *C. remanei* females, Baird (2002) demonstrated that sexual transformation is the cause of Haldane's rule in these species. Perhaps the relative neglect of this hypothesis stems from the fact that sex chromosomes are usually a good predictor of sex and that sexual transformations are demonstrably not the cause of Haldane's rule in so many taxa. Additionally, this hypothesis can only ever explain the absence of males, not their sterility.

### 4.2 Chromosomal rearrangements

In 1932, Haldane provided yet another hypothesis in his book ‘The Causes of Evolution’. This is based on evidence from *Drosophila* where an X‐Y translocation (where one section of a sex chromosome breaks off and is relocated on the other) being artificially induced resulted in sterile males because they lacked part of the Y chromosome. As Laurie (1997) points out, it is difficult to see how reciprocal translocation would become fixed in a population and the idea is essentially dismissed. However, the idea should not be completely dismissed as multiple movements between the Y chromosome and autosomes have been documented in *Drosophila* lineages, with all such events creating the potential for male‐specific problems in these hybrids (Koerich et al., 2008). Recent proof of translocations from autosomes to the sex chromosomes in *Rumex hastulatus*, which is only the second example of plants displaying Haldane's rule, also suggests that chromosomal rearrangements might be an interesting phenomenon in the plant kingdom regarding Haldane's rule (Kasjanuk et al., 2019).

### 4.3 Y incompatibilities

Another hypothesis that has been posited is that because Y (or W) chromosomes are largely heterochromatic, there may be Y‐X or Y‐autosome incompatibilities leading to the heterogametes being sterile (Muller, 1942). Pantazidis and Zouros (1988) showed that when *Drosophila arizonensis* males carry the Y chromosome of *D. mojavenis* they are sterile due to immotile sperm. However, they also found that when one of the fourth autosome pairs is replaced with a homologue of *D. arizonensis* as well, the sperm is motile once again. A Y‐autosome interaction is therefore causing this sterility. There have been discoveries of significant effects from the Y chromosome in other *Drosophila* species as well (Lamnissou et al., 1996), while other species show no such incompatibilities (Orr, 1989b). The fact that Y incompatibilities are implicated in cases of heterogamete sterility can be used to explain some instances of Haldane's rule for sterility and is therefore a good reason to expand this research into other taxa. The Y chromosome is not usually required for viability though, as evidenced by the large number of species with XO chromosomes that produce viable hybrid offspring (Voelker & Kojima, 1971). Haldane's rule for inviability therefore requires an alternative explanation.

### 4.4 Dosage compensation

Coyne and Orr (1989b) point out that a potential contributing factor to Haldane's rule is a breakdown in dosage compensation, the process by which the expression of sex‐linked and autosomal genes are balanced. In *Drosophila*, dosage compensation involves an increase in the transcription of the genes on the male X chromosome (Baker et al., 1994) (see Table 2). Coyne and Orr note that this could cause Haldane's rule in two ways, the first involving the cis‐acting sequences near the X‐linked genes diverging between the two species. This would result in one species' X‐linked genes not being recognized by the other species' regulatory sequences, wreaking havoc in the compensation process. The other way would involve problems with the hyperactivation of X owing to the counter genes on the autosomes (which either allow or disallow the hyperactivation of X) not being recognized as a result of divergence in the two species. Likewise, the number of these counter genes could also diverge. Orr (1989a) used *D. melanogaster* females and *D. simulans* males in a cross to test the hypothesis that a breakdown in dosage compensation is a cause of postzygotic isolation. Specifically, Orr made clever use of the *sxl* locus, which blocks or allows hyperactivation based on the X:Autosome ratio. By introducing this *sxl* control gene into sterile hybrid males, the dosage compensation is corrected and, if dosage compensation were to be the cause of sterility or inviability, the hybrids would become fertile. Orr's experiments did not yield this result and the hybrid males remained completely sterile or inviable, evidence which suggests that dosage compensation does not explain Haldane's rule.

**TABLE 2:** The sex determination system in *Drosophila*

	Number of X chromosomes	Number of autosome sets	X:Autosome ratio	Outcome
Male	1	2	0.5	Upregulation of the X
Female	2	2	1	No need for upregulation

While this result has convinced many that dosage compensation is not the cause of Haldane's rule, it should be noted that there is a myriad of different ways in which dosage compensation operates in taxa other than *Drosophila* (Marin et al., 2000) and therefore this hypothesis should be more widely explored. There have also been some interesting findings since 1989 which suggest that dosage compensation might cause Haldane's rule in conjunction with other hypotheses. These will be discussed in the faster‐X section of the review.

### 4.5 Dominance theory

Of all the theories that seek to explain Haldane's rule, the dominance theory likely enjoys the most support and readily provides good evidence that can easily be linked to the BDM incompatibility example presented earlier, originally from Dobzhansky (1936). However, the history of this theory is fraught, beginning with it being verbally proposed by Muller, only to be rejected experimentally 50 years later, before finally being resurrected with a few alterations (Orr, 1997).

**FIGURE 1:**
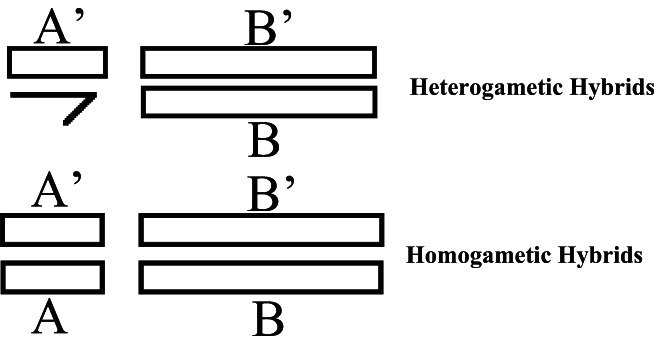
Autosomes are represented by the long bars, which have both dominant (*B′*) and recessive (*B*) alleles. The same goes for the homogametic sex chromosomes, which have dominant (*A*) and recessive (*A*′) alleles. However, note the absence of the dominant allele in the heterogametes sex chromosomes.

The best way to explain this is to imagine two autosomal genotypes in two different species, one with genotype *A*′*A*′*BB* and the other with genotype *AAB*′*B*′ such that when they interbreed, their offspring have genotype *A*′*AB*′*B*. Imagine now that the *A*′ locus is X‐linked, and the *B*′ locus is on an autosome (Muller, 1942). The male genotype will be *A*′*B*′*B* (where one allele is missing due to only one X chromosome being present). If there were any harmful interactions between *A*′ and *B*′, and *A*′ was recessive while *B*′ was dominant, then the dominant *A* allele's absence would not mask the effects of this harmful interaction, whereas the female genotype of A'AB'B would (see Figure 1 for a visual representation of this). The key point to understand here is that there is an epistatic interaction that is exposed in heterogametes but not in homogametes because the latter experiences a dominance interaction. A scenario such as this will give rise to Haldane's rule.

With Muller's theory being known, Coyne (1985) sought to test his theory experimentally. Muller posited that if Haldane's rule is due to the recessivity of X‐linked genes then females with two identical X chromosomes should also experience sterility and inviability. Coyne tested this with crosses in *Drosophila* species where an X chromosome from *D. simulans* was attached to an otherwise hybrid genetic background with haploid autosomes from other *Drosphila* species: *D. sechelia* and *D. mauritiana*. These attached X chromosomes produced an unbalanced hybrid female that was expected to be sterile. However, all unbalanced females turned out to be perfectly fertile. Muller's explanation therefore does not hold in the case of male hybrid sterility.

Coyne (1985)'s experiment did not lead to the complete dismissal of Muller's dominance theory. Wu (1992) subsequently noted that the theory may still hold true for the inviability of the heterogametic sex. Tests for inviability soon followed from Orr (1993a) who used *D. simulans* females with an attached X chromosome, to cross with *D. teissieri* males, which produced females that are genetically as unbalanced as inviable hybrid F1 males. Orr found these females (as well as males) to be lethal and also revealed that these unbalanced females died during the same developmental stage as the male hybrids, strongly suggesting that the same loci cause lethality in males and unbalanced females. Orr also did a species cross between female *D. melanogaster* with an attached X chromosome and a male *D. simulans* and, again, both sexes were lethal. What can be concluded from research from both Coyne (1985) and Orr (1993a) is that when it comes to Haldane's rule, the causes of sterility and inviability are different because factors affecting fertility have sex‐limited effects, whereas those affecting viability do not.

Following Orr (1993a)'s experimental evidence, Muller's theory was promptly formalized mathematically (Orr, 1993b; Turelli & Orr, 1995). A key finding of Orr's (1993b) model was that if Muller's original theory were true, and the X‐linked genes are masked in hybrid females, then the females would have two of these incompatibility genes and would surely suffer from the additive effect of carrying twice as many, causing the forces acting in both hybrid sexes to balance, leading to both suffering equally. Orr therefore modified Muller's theory slightly with a new condition that states that these X‐linked speciation genes must be at least slightly recessive on average. Evidence that the loci contributing to isolation are at least partially recessive is therefore needed as proof. Handily, this proof had already been supplied decades earlier by Muller and Pontecorvo (1942) and subsequent introgressions, the repeated backcrossing of hybrids also proved this (True et al., 1996). Presgraves (2003) approached the test differently by causing small chromosomal deletions in *D. melanogaster* and then using a rescue mutation from *D. simulans* to produce hybrid offspring that were hemizygous for the deleted regions. Presgraves discovered a number of incompatibilities between these two species and estimated that the recessive incompatibilities are eightfold higher in number than dominant ones.

As evidence has accumulated in support of the dominance theory there has at times been a tendency to dismiss the other hypotheses altogether. Indeed, Laurie (1997) felt the need to specifically warn against doing so. A note of hesitancy is of course warranted when almost all evidence for the dominance theory came from the *Drosophila* genus. There is further evidence of dominance theory from *Anophele*s mosquitos, but these have the same X‐Y, male heterogametic system as *Drosophila* (Slotman et al., 2007). However, cases of Haldane's rule in the female heterogametic system of Lepidopterans now point towards dominance theory as at least one of the possible explanations of isolation between species in this taxon (Davies et al., 1997; Salazar et al., 2005).

There is, however, a taxon in which dominance theory can be conclusively ruled out: marsupials. In a crucial paper, Watson and Demuth (2012) point out that female mammals inactivate one of their X chromosomes so that it is transcriptionally silent, making them functionally hemizygous. They note that placental mammals differ from marsupials in that the former randomly inactivates either the paternal or maternal X chromosomes, whereas the latter will always inactivate the paternal sex chromosome. They explain that the consequences of this are that placental mammals produce a mosaic of maternal and paternal X chromosomes, while the marsupials have the consistent hemizygous expression of the maternal X chromosome. This led them to postulate that Haldane's rule should not hold true in marsupials if it is to be explained by dominance theory, because the hybrid males and females are expected to suffer equally in the face of X‐linked incompatibilities. However, they note that Haldane's rule is observed in marsupials and so an alternative explanation is needed.

### 4.6 Faster‐male theory

A well‐supported explanation that can also account for Haldane's rule is the so‐called faster‐male theory, as proposed by Wu and Davis (1993). They point to evidence that shows male reproductive characters evolve at a faster rate than female ones. This is attributed to the selective pressures on males being particularly strong, owing to intra‐ and intersexual selection, which is less strong on females. They also point to a number of differences between spermatogenesis and oogenesis, such as the lack of postmeiotic transcription in the former, and suggest that this may cause the process to be more sensitive to minor stoichiometric changes in the gene products as there is no capacity for the regulation of transcription.

Malone and Michalak (2008) find good evidence that oogenesis can tolerate a great deal of gene misexpression in a cross between the frog species *Xenopus laevis* and *X. muelleri*. They used these frogs because they are a female heterogametic system that is a rare exception to Haldane's rule. It was found that despite hybrid females being perfectly fertile, they have far greater gene misexpression compared with the hybrid males, suggesting that the process of oogenesis is indeed less easily disturbed. The fact that *Xenopus* frogs are a female heterogametic system means that, despite these frogs not conforming to Haldane's rule, they provide rather good support for the faster‐male hypothesis in male heterogametic taxa if the same mechanism is at work. Evidence that this is the case in male heterogametic systems comes from the fragility of spermatogenesis in crosses between the nematodes *C. briggsae* and *C. nigoni*, which exhibit Haldane's rule (Sanchez‐Ramirez et al., 2021).

Faster‐male evolution also appears to have been supported by a collation of data on crosses in *Anopheles* and *Aedes* mosquitos (Presgraves & Orr, 1998), as well as introgression studies in *Drosophila* (Masly & Presgraves, 2007; Tao & Hartl, 2003), *Hyla* frogs (Dufresnes et al., 2016) and *Cyprinodon* fishes (Tech, 2006). Identifying the specific genes involved in the misregulation of spermatogenesis was also shown to be possible by Gomes and Civetta (2014) who found that sterility specific to male hybrids was driven by three genes in *Drosophila*: *pelo*, *vis* and *topi*.

Somewhat unorthodoxly, hermaphroditic species have also been the subject of research in the 21st century. Although simultaneous hermaphrodites lack distinct sexes, the functionality of the male and female genitalia can be used to identify instances of Haldane's rule and assess whether faster‐male evolution is taking place. This is because, if faster‐male evolution is causing Haldane's rule, then this should occur in the absence of sex chromosomes as well (Orr & Presgraves, 2000). Schilthuizen et al. (2011) found just this by reviewing the cases of defects in hybrid hermaphrodites in Pulmonata. They found that genital defects were rare but predominantly found in male genitalia.

Faster‐male theory would serve as an explanation for why Haldane's rule was observed by Coyne (1985) and Watson and Demuth (2012) where males were the heterogametic sex and dominance theory could be ruled out as an explanation. However, there are some issues with this theory. Most obviously, Haldane's rule does not refer specifically to males, but to heterogametes such that, as noted by many previous reviewers of the subject, it can only ever be a partial explanation of Haldane's rule and it should in fact work contrary to Haldane's rule in ZW sex determination systems, as is the case in the aforementioned *Xenopus* frogs of Malone and Michalak (2008) (Coyne & Orr, 2004; Delph & Demuth, 2016; Laurie, 1997; Orr, 1997). A further sticking point is that this theory only accounts for Haldane's rule for sterility, not inviability as the lethal mutations will usually kill both sexes (Hollocher & Wu, 1996; Wu et al., 1996). It looks to be the case then that the faster‐male theory can explain instances of Haldane's rule that dominance theory cannot by itself account for.

### 4.7 Faster‐x theory

Yet another explanation of Haldane's rule was posited by Charlesworth et al. (1987) who suggested that it could be a by‐product of the other rule of speciation: the large‐X effect, which refers to the disproportionately large role of the X chromosome in reducing hybrid fitness. This original paper suggests that both these rules of speciation could be explained by X (or Z) linked genes evolving faster than autosomal ones. Presgraves and Meiklejohn (2021) point out that this assumes that unique beneficial mutations that are fixed in the population are the basis for adaptation, that there are equal germline mutations for the two sexes and that there is an equal sex ratio, implying that the effective population size of X is ¾ that of autosomes. They note that the ratio of the rate of adaptive substitution on the X chromosome and autosomes varies depending on the dominance coefficient of a given mutation (dominance within the species in this case, not in the hybrids). Therefore, if natural selection is the force driving evolution, then only mutations that are on average partially recessive will experience faster‐X evolution. The relevance of these recessive mutations is that if X‐linked incompatibilities increase in frequency they will favour the occurrence of Haldane's rule if they are recessive, while the opposite is true if this is not the case (Turelli & Orr, 1995). This is because the heterogametic sex suffers less than the homogametic sex when X‐linked dominant mutations are concerned, owing to the fact that the former only has one X chromosome. However, if the mutation is recessive, then the heterogametes suffer in the absence of the heterozygosity present in the homogametes (Laurie, 1997).

Orr (1993b) points out the problem with the faster‐X theory: it cannot explain Haldane's rule by itself because, even if all alleles are recessive and the X chromosome evolves faster than the autosomes, Haldane's rule will not be observed. Orr notes that this is because every recessive X‐linked allele from a male will be accompanied by a dominant allele in a female. Orr (1997) explains that, instead of faster‐X causing Haldane's rule per se, under certain circumstances, the faster‐X theory can work alongside the other explanations of Haldane's rule to increase isolation. The first scenario considered is that the genes causing hybrid sterility or inviability may also be recessive in hybrids, causing the mechanism on which the dominance theory is based to be exaggerated. The second is that many genes that affect hybrids might only be expressed in one sex, leading to faster evolution of the genes expressed in the heterogametic rather than the homogametic sex when advantageous new mutations are partially recessive (Coyne & Orr, 1989b).

As with the other hypotheses, introgression studies have been used to test the faster‐X hypothesis. An example is the introgression of X‐linked genes across hybrid zones between the mouse subspecies *Mus musculus musculus* and *M. musculus domesticus*. (Payseur et al., 2007). However, it is important to consider what exactly this evidence supports. As Delph and Demuth (2016) point out in their review, this is a demonstration of the large‐X effect, but not necessarily evidence of faster‐X as an explanation of Haldane's rule. Good evidence comes in the form of genome‐wide studies, which show that faster‐X evolution occurs in sex chromosomes of *Drosophila* relative to autosomes (Begun et al., 2007). Interestingly the earlier findings in *Drosophila* found the opposite (Betancourt et al., 2002), so mounting evidence, coming from genome‐wide techniques, is building in favour of faster‐X being at least somewhat important in explaining Haldane's rule. Faster rates of evolution also exist on the X chromosome of Aphids than in their autosomes (Jaquiery et al., 2018) and Z chromosomes also evolve faster in birds (Mank et al., 2007) and Lepidopterans (Sackton et al., 2014). Beyond these individual studies, reviews of the large‐X effect show that sex chromosomes being more differentiated than autosomes is a significant and widely observed pattern (Presgraves, 2018).

Most recently, Filatov (2018) reported on the role of degeneration of the Y chromosome and how this can potentially drive compensatory evolution of dosage compensation mechanisms on the X chromosome, leading to faster‐X evolution. Filatov's work makes use of data from plant crosses in *Silene*, which exhibit Haldane's rule and have Y chromosomes that degenerated rapidly but rather recently compared with most animals, alluding to the importance of hemizygosity being reduced in plants relative to animals when it comes to explaining Haldane's rule. Filatov's findings serve as a potential explanation for both rules of speciation, and it should be noted that, because this explanation involves dosage compensation, his findings add to the evidence that dosage compensation, previously discussed, could play an important role in postzygotic isolation. Filatov also notes that this explanation is unlikely to be relevant to animals as their Y chromosomes are older and degenerate at a slower rate. However, it is worth noting that taxa lacking dosage compensation, such as Lepidopterans, are less likely to be affected by faster‐X evolution suggesting that there is some association between this hypothesis and dosage compensation (Presgraves, 2002).

### 4.8 Meiotic drive

A further theory, first proposed by Frank (1991) and Hurst and Pomiankowski (1991), with a history of fluctuating levels of support, is that divergence between the meiotic drive systems between species could be responsible for Haldane's rule. Meiotic drive can be defined as a distortion of mendelian ratios by selfish genetic elements (Toothill, 1984). Frank's (1991) argument consists of two key points: the first is that sex chromosomes experience greater levels of meiotic drive than autosomes and the second is that the systems of meiotic drive diverge between species which leads to postzygotic isolation.

Frank (1991) explains the former by verbally detailing a scenario where the drive is caused by the interaction between two loci on the X chromosome that would increase the reproductive success of the X chromosome at the expense of the Y chromosome, with the same being true of alleles on the Y chromosome. Frank then supposes the existence of a distorter allele on an autosome that biases segregation by producing a product that destroys a responder allele. If the distorter and responder are on the same chromosome, the distorter will destroy its own chromosome and so it is only under rare circumstances that cooperating loci are found on autosomes, leading to more meiotic drive on sex chromosomes. Frank then explains the second point by imagining a number of co‐evolved suppressors and distorters on the sex chromosomes where they each cancel out so that no drive occurs. In hybrids, however, no such co‐evolution has occurred as the chromosomes originate from different species and so are sterile due to meiotic aberrations or inviable due to mitotic aberrations.

Following the proposal of this theory, it was promptly pointed out that there was no empirical evidence for it. In fact, Johnson and Wu (1992) and Coyne and Orr (1993) showed that, in *Drosophila* hybridisations, there were no signs of meiotic drive. In his 1997 review on Haldane's rule, Orr roundly dismisses meiotic drive as an explanation claiming that it had been proven not to be the cause of Haldane's rule and that, at best, it may act now again. By 2004, this dismissiveness had all but vanished and Coyne and Orr (2004) dedicated no less than three pages in their book on speciation to considering the faults and merits of this theory (faster‐X by comparison received only one page). They suggested that the previous studies were unfortunate in their choice of species and that some species of *Drosophila* do in fact show meiotic drive. For example, Tao et al. (2001) found a small, tightly linked region on chromosome three of *D. simulans* that reduced hybrid male fertility and in doing so caused meiotic drive. This was a dominant conspecific suppressor named *Tmy* and when one of these was replaced with a recessive nonsuppressing allele (*tmy*) from *D. mauritiana*, the dominance of *Tmy* caused the sex distortion expected under a meiotic drive scenario.

Coyne and Orr (2004) are quick to point out, however, that this is not in and of itself proof that meiotic drive is the force behind postzygotic isolation. Instead, they point to two scenarios that could fit with the aforementioned findings. One is that the meiotic drive arose in two species and are incompatible with each other when they mate to produce hybrids, the other is that meiotic drive never actually occurred in either species. These two scenarios are indistinguishable from one another.

However, important pieces of evidence in support of meiotic drive are being pieced together, including sex chromosome drive systems in *Caenorhabditis* where X‐carrying sperm outcompete Y‐bearing sperm (Bundus et al., 2015) and proof of driver/suppressor systems evolving rapidly (Presgraves et al., 2009). Patten (2018) recently reviewed some of the evidence supporting meiotic drive as a cause of hybrid incompatibility and concluded that genetic conflicts commonly co‐evolve and that empirical evidence now supports this hypothesis as a potential explanation of Haldane's rule in many cases. Patten notes, however, that an expansion beyond the common model organisms would make a much more compelling case for this theory.

## 5 CONCLUSIONS

100 years have now passed since J.B.S Haldane made the famous observation that came to be known as Haldane's rule. During this time, what was a phenomenon observed only in a few taxa has spread to ever more animal taxa and even into the plant kingdom. The universality of Haldane's rule is now almost unanimously accepted despite, and in some ways thanks to, Read and Nee's (1991) intervention.

The approaches that have been employed to study the causes of Haldane's rule have also changed over the years. As ever more is understood about the genetics of speciation, it has become clear that there are multiple causes of Haldane's rule. Sometimes there is a single cause, in other cases they act together (dominance and faster‐X theory for example). This being said, dominance theory and faster‐male theory have emerged as the primary explanations of Haldane's rule. It is important to bear in mind though that neither of these hypotheses is applicable in all sex determination systems. After 100 years of research, the less well‐explored hypotheses could still prove to be very important to the understanding of Haldane's rule. Meiotic drive looked at one time to have been falsified but now appears to be an interesting explanation that would benefit greatly from more empirical study. It may be a mistake to perform this research only in *Drosophila* species and the expansion into ever more unorthodox taxa is something that must continue to occur if this general rule is to be understood. Returning to the comparative approach that Haldane (1922) took in order to make his initial observation is therefore still necessary today.

## CONFLICT OF INTEREST

No conflicts of interest to declare.

### PEER REVIEW

The peer review history for this article is available at https://publons.com/publon/10.1111/jeb.14112.

## Data Availability

The manuscript contains no new data.
